# Immunometabolic Organ Crosstalk in Heart Failure with Preserved Ejection Fraction: The Role of Dietary Patterns in Obesity-Related Inflammation

**DOI:** 10.3390/nu18111720

**Published:** 2026-05-28

**Authors:** Małgorzata Kalisz, Anna Litwiniuk, Natalia Sławkowska, Dominika Stępień, Wojciech Bik

**Affiliations:** Department of Neuroendocrinology, Centre of Postgraduate Medical Education, Marymoncka 99/103, 01-813 Warsaw, Poland; alitwiniuk@cmkp.edu.pl (A.L.); nslawkowska@cmkp.edu.pl (N.S.); dstepien@cmkp.edu.pl (D.S.); wbik@cmkp.edu.pl (W.B.)

**Keywords:** heart failure with preserved ejection fraction, organ crosstalk, dietary patterns, inflammation

## Abstract

Heart failure with preserved ejection fraction (HFpEF) is a major healthcare problem affecting approximately 1–2% of the adult population in highly developed countries. It is a heterogeneous condition with cardiometabolic disorders such as obesity, insulin resistance, diabetes mellitus, and hypertension. Pathophysiologically, HFpEF is currently recognized as a systemic disease characterized not only by impaired left ventricular relaxation and increased ventricular stiffness but also by chronic inflammation, endothelial dysfunction, myocardial fibrosis, and multi-organ involvement. This study aims to investigate the role of metabolic organ crosstalk and nutritional modulation in different HFpEF phenotypes. This review analyzes the current literature to investigate the interactions between the heart, adipose tissue, liver, and spleen in the pathophysiology of HFpEF. Chronic low-grade inflammation and adipose tissue dysfunction play central roles in the progression of HFpEF by activating the immune system, favoring ectopic lipid accumulation, and exacerbating fibrosis. The identification of specific HFpEF phenotypes (e.g., obesity-related versus aging-related) requires the application of distinct nutritional strategies that target metabolic inflammation and organ crosstalk, which may improve both myocardial and systemic function. HFpEF is a complex systemic disorder that requires phenotype-specific therapeutic approaches. Precision nutrition based on specific biomarkers, together with comprehensive cardiovascular management, may enhance therapeutic efficacy and complement pharmacological treatment in patients with HFpEF.

## 1. Introduction

In highly developed countries, heart failure (HF) prevalence is approximately 1–2% of the total adult population, and approximately 50% of those are heart failure with preserved ejection fraction (HFpEF) [1]. The global increase in cardiometabolic diseases, which is driven by unhealthy lifestyle factors and the growing prevalence of coexisting metabolic diseases, is therefore a significant factor in the increase in the incidence of HFpEF [2]. HFpEF is not a single condition, but the result of many different pathologies such as obesity, diabetes mellitus (DM), hypertension, and chronic kidney disease.

Initially, it seemed that the pathophysiology of HFpEF is caused only by left ventricular (LV) diastolic dysfunction. However, it is now recognized to encompass systemic abnormalities, including myocardial fibrosis, impaired cardiac contraction and relaxation, chronotropic incompetence, hypertension, skeletal muscle dysfunction, kidney impairment, and systemic inflammation [1]. It has been suggested that a central mechanism in HFpEF pathophysiology may be chronic inflammation, leading to endothelial dysfunction and cardiac abnormalities [3]. This wide range of biological disorders, in their clinical presentation, underscores the importance of integrating multiple metabolic organs to understand cardiac and extracardiac mechanisms and to identify the most suitable therapy for the HFpEF phenotype.

HFpEF is usually associated with obesity and insulin resistance (IR), which leads to adipose tissue (AT) dysfunction, increased inflammation, ectopic lipid deposition, and altered adipokine signaling, all of which adversely affect myocardial structure and function. Chronic inflammation activates the liver and spleen to produce inflammatory cells, which are then mobilized to the heart, leading to chronic inflammation, progressive fibrosis, and potentially decreased cardiac function. In metabolic and inflammatory disorders, nutrition represents a modifiable therapeutic factor. Therefore, nutritional strategies that modulate metabolic organ function represent a promising approach to addressing the complex nature of HFpEF and may complement pharmacologic therapies.

## 2. Pathophysiology of HFpEF

Despite optimal medical treatment, HFpEF is associated with a 40% mortality rate within 5 years of diagnosis [4,5,6]. The European Society of Cardiology (ESC) definition of HFpEF is as follows: “Symptoms and signs of HF, with evidence of structural and/or functional cardiac abnormalities and/or raised natriuretic peptides (NPs), and with a left ventricular ejection fraction (LVEF) ≥ 50%” [7]. Unlike heart failure with reduced ejection fraction (HFrEF), there is no single abnormal echocardiographic parameter that defines HFpEF, a heterogeneous clinical syndrome. It is also difficult to diagnose HFpEF because approximately 20% of patients, mostly with obesity, have normal NP levels [8]. Importantly, obesity and type 2 diabetes mellitus (T2DM) impair heart function and lead to metabolic disorders that contribute to increased inflammation, tissue fibrosis, and increased myocardial cell stiffness, all of which are key features observed in HF [9,10]. AT, recognized as an organ involved in regulating metabolic, hormonal, and immune responses, promotes lipotoxity, hypertrophy, and cardiac remodeling, ultimately leading to cardiomyocyte damage [11,12].

### 2.1. Role of Chronic Low-Grade Inflammation and Endothelial Dysfunction

HFpEF is characterized by chronic and low-grade inflammation. It occurs at both the systemic and myocardial levels [13]. It results in endothelial inflammation and coronary microvascular disorders, which limit nitric oxide (NO) bioavailability and the activity of protein kinase G (PKG) in cardiomyocytes. The resulting cardiomyocyte hypertrophy and fibrosis, together with reduced ventricular compliance, contribute to the pathogenesis of HFpEF [14]. Furthermore, inflammation in HFpEF is often attributed to comorbidities.

### 2.2. Phenotypes of HFpEF

HFpEF is a highly heterogeneous clinical syndrome that may present with various combinations of cardiovascular (CV), metabolic, pulmonary, renal, and geriatric conditions, which are estimated to be present in patients with HFpEF (Table 1) [15]. Identifying the most common HFpEF phenotypes and treatment strategies associated with each clinical profile appears to be the most promising approach to improving clinical outcomes.

#### 2.2.1. Obesity-Related HFpEF

Among all phenotypes, obesity-related HFpEF has received particular attention because of the central role of adipose tissue dysfunction in metabolic and inflammatory regulation. Patients with obesity-related HFpEF exhibit several characteristic CV irregularities, including increased pulmonary vascular resistance, elevated mean pulmonary arterial pressure, and evidence of right ventricular dysfunction, manifested by an increased right atrial-to-pulmonary capillary wedge pressure ratio and interventricular septal flattening [15].

#### 2.2.2. Age-Related HFpEF

However, although HFpEF is generally considered a disease of older adults, up to 40% of cases occur in patients younger than 65 years of age [15]. Younger patients are more often Black and Asian men with obesity and T2DM, lower comorbidity burden, and poorer quality of life, whereas older patients are predominantly white women with hypertension, atrial fibrillation, and chronic kidney disease. Older individuals experience higher overall mortality, driven predominantly by non-CV causes, while younger patients are more likely to experience CV mortality, particularly sudden cardiac death [15].

#### 2.2.3. Sex-Related HFpEF

Women are likely to be affected by HFpEF, not HFrEF, compared to men, due to sex-specific differences in genetics and epigenetics, smaller ventricular chambers and smaller vasculature, estrogen exposure, and signaling pathways for protection against oxidative stress and inflammation. However, after menopause, women typically exhibit greater metabolic risk factors (obesity, hypertension, diabetes, dyslipidemia), iron deficiency, higher prevalence of metabolic and autoimmune comorbidities, preeclampsia, breast cancer, and its treatment. Despite experiencing more severe symptoms and poorer quality of life, women with HFpEF are generally characterized by lower CV and non-CV mortality compared with men [15].

#### 2.2.4. Type 2 Diabetes Mellitus (T2DM)-Related HFpEF

T2DM is one of the most prevalent comorbidities in HFpEF, and its prevalence ranges from approximately 29% in Europe and 44–57% in the Asia-Pacific region [15]. In patients with HFpEF, this disease has been shown to independently increase the risk of hospitalization, all-cause mortality, and CV mortality.

#### 2.2.5. Frailty- and Cachexia-Related HFpEF

HFpEF is associated with older age, multiple comorbidities, reduced physical function, and a higher prevalence of sarcopenia and cachexia. These conditions may identify patients with more advanced or end-stage HFpEF and much higher mortality. However, their impact on treatment response remains poorly understood, although current evidence suggests that frailty does not diminish the benefits of SGLT2 inhibitors. Further research is needed to determine optimal therapeutic strategies and the potential role of advanced treatments in this vulnerable HFpEF phenotype.

## 3. Adipose Tissue Dysfunction in HFpEF

Importantly, the metabolic and inflammatory activity of AT differs between fat depots, which may influence cardiometabolic risk. Obesity, especially visceral adipose tissue (VAT) accumulation, is a common clinical phenotype of HFpEF [16]. This phenotype significantly worsens the patient’s prognosis. Obesity contributes to the development of HFpEF through increased blood volume, cardiomyocyte hypertrophy and fibrosis, inflammation, and impaired metabolic homeostasis. These mechanisms induce diastolic dysfunction and elevated LV filling pressures, both of which are characteristic symptoms of HFpEF [17].

AT functions as an endocrine organ that produces adipokines—bioactive proteins that mediate a wide range of metabolic, immune, inflammatory, and CV processes [18]. These mediators include polypeptides and proteins (e.g., leptin, adiponectin), steroids (e.g., aldosterone), lipokines (e.g., fatty acids (FAs)), metabokines (by-products of cellular energy metabolism), and adipoexosomes—nanosized extracellular vesicles that may contain proteins and microRNAs capable of regulating gene transcription in distant tissues [19]. Moreover, increased adipoexosome abundance is associated with adipocyte inflammation, which is observed in patients with obesity or HFpEF [20].

Visceral obesity and ectopic fat depots, particularly increased epicardial adipose tissue (EAT), are harmful because they act as metabolically active endocrine organs, increasing inflammation and potentially contributing to endothelial dysfunction, myocardial fibrosis, and ventricular remodeling [21].

AT inflammation induces structural and functional remodeling, such as adipocyte hypertrophy, hypoxia, increased extracellular matrix deposition, and immune cell infiltration. These modifications promote a shift from an anti-inflammatory M2 macrophage phenotype to a pro-inflammatory M1 macrophage phenotype. In obese individuals, hypertrophic adipocytes increase production of pro-inflammatory cytokines such as tumor necrosis factor-alpha (TNF-α), Interleukin-6 (IL-6), and Monocyte chemoattractant protein-1 (MCP-1). The accumulation of macrophages enhances local and systemic inflammation. It activates intracellular signaling pathways such as Nuclear Factor kappa-Light-Chain-Enhancer of Activated B cells (NF-κB) and c-Jun *N*-terminal kinases (JNK). Moreover, AT inflammation leads to systemic IR by disrupting insulin signaling in adipocytes and peripheral tissues. In addition, inflamed AT releases increased amounts of free fatty acids (FFAs) into the circulation, further exacerbating lipotoxicity and metabolic dysfunction. On the other hand, in people of normal weight, M2 macrophages predominate. Their activation helps prevent inflammation by increasing the secretion of the anti-inflammatory Interleukin-10 (IL-10) and reducing levels of the pro-inflammatory Interleukin-12 (IL-12) and Interleukin-23 (IL-23) [22,23,24].

### 3.1. Visceral vs. Subcutaneous Adipose Tissue

Subcutaneous adipose tissue (SAT) and VAT are two principal compartments of abdominal adiposity. Some studies suggest a link between elevated VAT and the risk of an unfavorable metabolic profile, regardless of total body fat [24]. VAT has also been shown to increase the risk of cardiometabolic diseases [25,26]. SAT, on the other hand, is considered a neutral fat storage area. There are also suggestions that SAT has a protective effect against T2DM and coronary heart disease (CHD) [24,27,28,29]. A systematic review, in turn, indicated that a higher VAT to SAT ratio in the abdomen may be associated with the development of cardiovascular disease (CVD) [30].

### 3.2. Obesity, Insulin Resistance, and Myocardial Stiffness

Cardiac IR, often associated with obesity, is defined as the inability of insulin to activate its signaling to effectively regulate cellular processes, including glucose uptake and utilization in the heart [31]. It is known that cardiac IR contributes to cardiac dysfunction [32]. Zhu et al. demonstrated that early-onset systemic IR in non-diabetic patients can disrupt the physiological function of EAT and lead to myocardial fibrosis [33]. Moreover, in patients with metabolic syndrome (MetS) and IR, abnormal EAT metabolism can exacerbate myocardial fibrosis [34]. Insulin receptors are widely expressed in the heart and vasculature, where they regulate cardiac growth and function, substrate uptake and utilization, mitochondrial metabolism, and the suppression of apoptosis and autophagy. Experimental studies suggest that a mouse model of cardiomyocyte insulin receptor deletion (CIRKO) may exhibit accelerated LV remodeling in response to pressure overload [35]. Impaired insulin signaling is also thought to contribute to pathological cardiac remodeling and myocardial stiffness, potentially promoting the development of HFpEF [36].

### 3.3. Pericardial and Epicardial Adipose Tissue and Cardiac Mechanics

EAT is located around the atrioventricular sulci, interventricular sulcus, coronary arteries, atria, free wall of the right ventricle, and apex of the left ventricle. Importantly, there is no anatomical barrier between EAT and the heart muscle. Furthermore, these structures share a common coronary microcirculation. Together, these features enable EAT to directly modulate myocardial metabolism, including providing FFAs and protecting the heart against excess circulating lipids [37,38]. EAT can contribute to the development and progression of HFpEF; its amount is typically increased and correlates with worse function/outcomes [39]. Quantification of EAT volume or thickness may provide clinically relevant information regarding cardiovascular and metabolic risk. Echocardiography has been the most widely used noninvasive imaging technique in clinical practice. This technique has some limitations; EAT thickness is usually measured in parasternal long-axis and short-axis views at end-systole, when the EAT layer is least compressed. Echocardiography provides limited information regarding total EAT volume and regional fat distribution. Multimodality imaging, compared to echocardiography, such as cardiac computed tomography (CT) and magnetic resonance imaging (MRI), enables more precise quantification of EAT distribution, volume, and thickness. CT using HU-based measurements allows for an objective quantification of EAT. Increased EAT attenuation provides important prognostic information for CVD and cardiac mortality. Novel imaging techniques, such as the use of 18F-fluorodeoxiglucose (18F-FDG) positron emission tomography (PET) is becoming more widespread, not only in the research setting but also for diagnosis of EAT’s inflammation, which may have implications in different cardiovascular processes [40].

Accumulation of EAT over the ventricles is associated with characteristic symptoms of HFpEF such as myocardial fibrosis, ventricular hypertrophy, and increased cardiac filling pressures and other markers of diastolic dysfunction [40]. These associations may be explained by the pro-inflammatory and profibrotic activity of EAT, which can contribute to myocardial remodeling through paracrine and systemic mechanisms.

EAT volume or thickness has gained attention as a potential therapeutic biomarker that may reflect the effectiveness of lifestyle interventions and pharmacological therapies, including weight reduction strategies, sodium-glucose cotransporter-2 (SGLT2) inhibitors, and glucagon-like peptide-1 receptor agonists. Therefore, further investigation will define the pathophysiological cause-and-effect relationship between the transformation and increase in EAT.

Pericardial adipose tissue (PAT), on the other hand, is located between the visceral and parietal pericardium. Unlike EAT, pericardial fat is the outer fat accumulation of the heart [41]. Excessive PAT deposits compress the heart muscle, reducing elasticity and remodeling, which can lead to diastolic dysfunction [42].

### 3.4. Systemic Inflammation and Microvascular Dysfunction

Microvascular dysfunction of the coronary vessels is recognized as a new element linking EAT and HFpEF [14,43,44,45,46]. It has been shown that HFpEF is associated with reduced microvascular density in coronary vessels, which is linked to myocardial fibrosis [47]. Some studies have shown that EAT surrounding the left ventricle correlates with the mean coronary flow reserve, which affects diastole [48]. Infiltration by activated macrophages thereby causes inflammation in cardiac tissues. Oxidative stress is also considered a key factor in the development of microvascular dysfunction of the coronary vessels [14,49]. When coronary microvascular endothelial cells produce excessive amounts of reactive oxygen species (ROS), the bioavailability of NO in adjacent cardiomyocytes is reduced. This process impairs the cGMP/PKG pathway, which under physiological conditions limits cardiomyocyte hypertrophy. Consequently, progressive ventricular hypertrophy and remodeling develop, leading to impairment of cardiac diastolic function [14,45].

### 3.5. Lipotoxicity and Altered Myocardial Energetics

In the context of obesity and IR, excessive lipolysis increases FFA concentrations, which accumulate in cardiomyocytes and promote cardiac lipotoxicity [50]. Moreover, FAs oxidation leads to increased ROS production and mitochondrial dysfunction. This is followed by cell apoptosis, driven by increased sphingolipid ceramide synthesis, which induces NO synthase [51]. This mechanism causes cardiac remodeling, including fibrosis. In patients with obesity, cardiomyocyte hypertrophy, extracellular matrix lipid infiltration, and pericardial fat accumulation are observed. These factors accelerate the progression of HFpEF [52,53].

## 4. The Role of the Spleen in HFpEF

The spleen is a highly vascularized immune organ with a crucial role in CVD [53]. It consists of red pulp (RP), white pulp (WP), and the marginal zone (MZ), which coordinate innate and adaptive immunity [54]. RP contains highly phagocytic macrophages, which remove aged and damaged erythrocytes and platelets [55,56]. WP contains T-cell–rich periarteriolar lymphoid sheaths (PALS) and B-cell follicles with dendritic cells and germinal center macrophages [57,58]. MZ macrophages interface innate and adaptive responses by capturing blood-borne antigens [58,59]. This structure ensures rapid activation of the immune system and systemic regulation of inflammation.

### 4.1. Splenic Monocyte Reservoir and Inflammatory Activation

The spleen serves as a major reservoir for Ly6C^high monocytes localized in the subcapsular RP [55,60]. Inflammatory or hemodynamic stress, including hypertension or myocardial injury, triggers angiotensin II–AT1R-dependent mobilization of these cells, distinct from CCR2–CCL2-mediated bone marrow egress [61]. Mobilized monocytes migrate to the injured myocardium, differentiating into inflammatory and reparative macrophages that coordinate tissue repair and modulate local inflammation [55]. While acute recruitment is protective, persistent monocyte activation contributes to chronic inflammation and fibrosis.

Splenic monocytes also influence systemic pathology by contributing to tumor-associated macrophage formation, atherosclerotic lesions development, and liver fibrosis [62,63,64], illustrating the spleen’s role as a regulator of systemic immune responses.

### 4.2. Neuroimmune Interaction in CVD

Autonomic innervation regulates splenic immunity. In detail, sympathetic fibers release norepinephrine, which acts on β_2_-adrenergic receptors on lymphocytes and macrophages, enhancing splenic contraction, monocyte mobilization, and hematopoiesis during CV stress [60,65]. Parasympathetic modulation occurs via the cholinergic anti-inflammatory pathway, in which acetylcholine binds to the α7 nicotinic acetylcholine receptor, thereby suppressing proinflammatory cytokines [66]. Cytokines such as Interleukin-1β (IL-1β) activate vagal circuits, thereby establishing a bidirectional neuroimmune loop [67]. It is worth noticing that dysregulated autonomic–splenic signaling perpetuates inflammation in HFpEF and HFrEF [68,69].

### 4.3. Splenic Involvement in Chronic Heart Failure: Evidence from HFpEF and Related Inflammatory Conditions

Systemic metabolic stress in HFpEF activates splenic macrophages and remodels the hematopoietic stem cell (HSC) niche, unlike the primarily myocardial inflammation in HFrEF [70]. Red-pulp macrophages upregulate Vascular Cell Adhesion Molecule-1 (VCAM-1) through FA-driven mitochondrial and epigenetic mechanisms, thereby enhancing HSC retention and proliferation. Elevated endothelial and myeloid VCAM-1 facilitates inflammatory cell trafficking, while myeloid-specific VCAM-1 suppression reduces splenic hematopoiesis [44,71,72,73].

Splenic macrophages in HFpEF adopt a hybrid inflammatory–reparative phenotype, TNF-α, IL-1β, IL-10, Cluster of Differentiation 206, and accumulate lipids that drive IL-23, Macrophage Colony-Stimulating Factor, and Granulocyte Colony-Stimulating Factor secretion, promoting HSC expansion [74,75]. Dysregulated FAs handling is reflected in elevated circulating long-chain acylcarnitines, a process that is mitigated by SGLT2 inhibitors through CD36 modulation and GMP suppression [76].

### 4.4. Contribution to Systemic Inflammation and Fibrosis

The spleen plays a key role in HFpEF-associated myocardial fibrosis. Ly6C^high splenic monocytes infiltrate the myocardium, differentiating into macrophages that secrete IL-1β, IL-6, Transforming Growth Factor- β, and Matrix Metalloproteinases, which activate fibroblasts, enhance collagen cross-linking, and increase diastolic stiffness [13,77]. The C-X-C Chemokine Receptor type 4 (CXCR4)-dependent mobilization amplifies cardiac macrophage accumulation, while CXCR4 blockade reduces fibrosis and improves diastolic function [78].

In summary, splenic monocyte trafficking and profibrotic reprogramming constitute a major extramyocardial mechanism in HFpEF, linking systemic inflammation, immune dysregulation, and myocardial stiffening.

## 5. Liver Dysfunction and HFpEF

Liver dysfunction often coexists with HFpEF. Liver impairment can exacerbate the CV mortality and hospitalization risks, while hepatic congestion caused by elevated right-sided filling pressures can further worsen liver function [79,80].

Congestive hepatopathy, associated with cardiocirculatory dysfunction, is characterized by sinusoid dilation, centrilobular fibrosis, and hepatocyte hypoxia. This condition promotes low-grade, inflammation-related activation of hepatocytes and nonparenchymal cells, including hepatic stellate cells, endothelial cells, and macrophages [81]. These cells form a vascular network that regulates blood flow and the filtration efficiency [82]. Injury to hepatocytes and cholangiocytes contributes to inflammation and fibrosis, as well as dysregulation of normal liver metabolic pathways, including glucose, lipid, amino acid, and bile acids (BAs) metabolism. Hepatic IR, impaired FAs utilization, systemic inflammation, altered hepatic urea cycle, and ammonia handling contribute to skeletal muscle dysfunction and exercise intolerance, which are hallmark clinical features of HFpEF.

### 5.1. Metabolically Associated Fatty Liver Disease

More than 50% of patients with HFpEF may also have non-alcoholic fatty liver disease (NAFLD) [83]. NAFLD and HFpEF share cardiometabolic risk factors, like obesity, DM, hypertension, and aging. It was demonstrated that NAFLD is associated with an increased incidence of CVD, including impaired cardiac structure and function, arterial hypertension, endothelial dysfunction, and early carotid atherosclerosis [83]. Evidence suggests that both conditions are manifestations of whole-body metabolism dysregulation rather than isolated organ dysfunction.

The secretion of adipokines and pro-inflammatory cytokines mediates the crosstalk between HFpEF and NAFLD. High leptin concentrations exhibit profibrotic activity by activating phosphoinositide 3-kinase, thereby enhancing osteopontin synthesis [84]. Additionally, it leads to cardiac hypertrophy and endothelial dysfunction. In AT, macrophages shift from an anti-inflammatory M2 phenotype to a pro-inflammatory M1 phenotype and secrete cytokines such as TNF-α and IL-6, which contribute to hepatocyte injury and NAFLD. Damaged liver cells produce Interleukin-33 (IL-33), which promotes a profibrogenic effect [85]. In the heart, IL-33 is released in response to myocyte stretch and promotes inflammation, myocardial fibrosis, and hypertrophy [86].

### 5.2. Hepatokines and CV Function

There are approximately 560 bioactive proteins, known as hepatokines, that are produced mainly by hepatocytes. These molecules may influence CV structure and function, including pathogenesis of HF, heart remodeling, microvascular inflammation, renal injury, and skeletal muscle myopathies [87]. Among hepatokines, adropin, fetuin-A, fetuin-B, fibroblast growth factor-21 (FGF-21), selenoprotein P, and α1-microglobulin regulate metabolic homeostasis and cardiac dysfunction in HF [87]. FGF-21 may exert a protective effect against cardiac hypertrophy by maintaining energy balance, increasing autophagy, reducing inflammation, oxidative stress, and apoptosis [88].

The BAs, long recognized for their role in lipid digestion, have recently emerged as signaling molecules that regulate metabolism and CV function [89]. In liver injury and NAFLD, BAs homeostasis is disrupted resulting in altered lipid accumulation and hepatic inflammation. Elevated concentrations of BAs in the heart may be cardiotoxic, leading to cardiomyopathy [90]. There are two main BAs receptors, the farnesoid X receptor (FXR) and Takeda G protein-coupled receptor 5 (TGR5)/GPBAR1, expressed in a wide range of tissues. Animal and clinical studies suggest that FXR agonism improves atherosclerosis [91,92]. In a mouse model of cholestatic liver disease induced by 3,5-diethoxycarbonyl-1,4-dihydrocollidine, cardiac hypertrophy has been observed and was suggested to be associated with overactive TGR5–Akt signaling [93].

## 6. Inter-Organ Crosstalk: Heart–Adipose Tissue–Spleen–Liver Axis

Metabolic factors, cytokines, adipokines, hepatokines, neurohormones, and immune cells mediate crosstalk among the heart, adipose tissue, spleen, and liver. The spleen-liver axis contributes to obesity-induced immune dysregulation, which is prominent in the comorbidities associated with HFpEF.

Systemic inflammation and excessive lipolysis elevate FFAs, disturbing cardiac function through structural remodeling, inflammation, and metabolic imbalance. Structurally, increased blood volume and myocardial wall stress contribute to LV hypertrophy and impaired diastolic dysfunction. Inflammatory cytokines drive fibrosis of cardiomyocytes via immune cell infiltration and collagen accumulation. Metabolically, elevated FFAs may infiltrate the cardiac extracellular matrix (ECM), which comprises type I and III collagen and fibronectin, thereby significantly influencing ECM stiffness and contributing to myocardial remodeling and diastolic dysfunction [94]. Furthermore, elevated FFAs concentration leads to mitochondrial dysfunction and energy imbalance in cardiomyocytes [95].

The expansion of AT, a metabolically active organ, promotes hypoxia, macrophage infiltration, and production of pro-inflammatory cytokines. Additionally, obesity leads to increased numbers of neutrophils, mast cells, and subsets of dendritic cells, further increasing the influx of pro-inflammatory cells [96]. Moreover, excessive lipolysis in AT contributes to the release of high levels of FFAs, leading to their accumulation in the liver and heart and inducing lipotoxicity, mitochondrial dysfunction, and oxidative stress. Increased myocardial FFAs uptake leads to overexpression of fatty acid transport protein 1 and lipotoxicity-mediated cardiomyopathy [97]. These processes promote structural remodeling of the heart.

High concentration of circulating FFAs ameliorates hepatic steatosis. Mechanistic studies indicate that increased uptake of FAs and de novo lipogenesis contribute to excessive intracellular accumulation of FFAs. These processes are accompanied by dysfunction in FA oxidation and lipid export from the liver [98]. It was confirmed that FFAs are toxic, and liver triglycerides (TG) accumulation may play a protective role. Inhibition of diacylglycerol acyltransferase 2 in the liver of a mouse model of T2DM and obesity on a severe nutritional deficiency diet may help prevent hepatic steatosis. Still, it led to increased ROS, inflammation, and liver fibrosis [99]. The liver, in turn, regulates systemic metabolic and CV homeostasis through the production of hepatokines and the regulation of lipoprotein and glucose metabolism.

The spleen plays an important role in regulating the immune system within this axis. It has been shown that splenic monocytes are involved in cardioprotective mechanisms following ischemic myocardial injury by enhancing their motility, migrating from the spleen, accumulating in injured tissue, and participating in tissue repair [55]. In the liver, during NAFLD, inflammation was associated with increased numbers of splenic myeloid-derived suppressor cells and natural killer T cells. In contrast, hepatic T- and B-cells loss was not reflected in the splenic lymphocyte landscape. Persistent low-grade inflammation, characteristic of obesity, sustains splenic activation, reinforcing systemic immune dysregulation.

**Figure 1:**
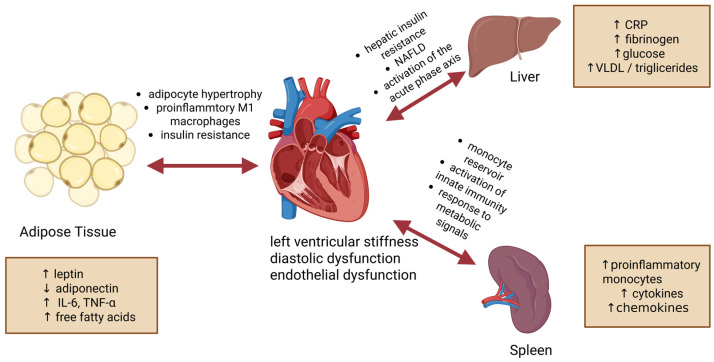
Heart–adipose tissue–spleen–liver axis. Created in BioRender. Kalisz, M. (2026) https://BioRender.com/4f1seic (accessed on 19 March 2026). Agreement number: GY29HQYHKN. ↑ indicates an increase; ↓ indicates a decrease.

Collectively, the heart–adipose tissue–spleen–liver axis operates through crosstalk involving metabolic regulation and immune activation, IR, oxidative stress, neurohormonal activation, and chronic inflammation as common mechanisms linking these organs (Figure 1).

## 7. Nutritional Patterns and Their Impact on HFpEF

It is important to understand that in patients with HFpEF, metabolic diseases comprising hypertension, DM, and obesity often coexist. Paradoxically, these patients also frequently suffer from malnutrition, which leads to worsening of their clinical state. In these patients, upregulated catabolic processes combined with reduced appetite contribute to metabolic imbalance [100].

### 7.1. Western Diet and Ultra-Processed Foods

Ultra-processed foods are characterized by extensive industrial processing and typically contain high levels of sugars, sodium, and trans fats, while being low in fiber. Such foods play an important role in disrupting glycemic control and insulin signaling [101]. Moreover, this type of food is generally a poor source of anti-inflammatory bioactive compounds, such as polyphenols [102]. A pilot study conducted in Australia in 2025 reported that high consumption of ultra-processed food was associated with a 19% higher risk of CVD-related mortality [103]. Notably, ultra-processed foods may also increase ROS production and oxidative stress, thereby activating pro-inflammatory pathways [104].

Overall, high consumption of ultra-processed food has been associated with obesity, hypertension, dyslipidemia, and gastrointestinal disturbances, subsequently leading to CVD, the development of MetS, and higher risks of cancer incidence [105].

### 7.2. Mediterranean Diet

The Mediterranean diet shares many features with the DASH (Dietary Approaches to Stop Hypertension) diet, such as a low intake of red meat and processed foods and the extensive use of extra-virgin olive oil (EVOO). EVOO is a great source of anti-inflammatory, unsaturated fatty acids. This includes monounsaturated FAs (MUFAs) and polyunsaturated FAs (PUFAs), which positively influence glucose and lipid metabolism [100].

It has been shown that the Mediterranean diet is more effective than a low-fat diet in preventing CVD [106,107]. A recent study showed that omega-3 FAs improved health parameters, including 6 min walking test results and VO2 peak in patients with HFpEF [108]. On the other hand, omega-3 supplementation may increase the risk of atrial fibrillation [100].

### 7.3. Plant-Based and Vegetarian Diets

Plant-based diets include large amounts of dietary fiber, antioxidants (primarily polyphenols), and healthy fats. Fiber, found in plants, helps slow glucose absorption and improve overall glucose management. Many polyphenols, in addition to their antioxidant potential, exhibit a wide range of therapeutic activities, including anti-inflammatory, anti-cancer, anti-diabetic, antimicrobial, cardioprotective, and neuroprotective effects [109].

Stavitz and colleagues demonstrated that this dietary pattern benefits patients with MetS by increasing insulin sensitivity, suppressing inflammation, and enhancing cardiovascular health through improved lipid metabolic homeostasis [110]. According to the researchers, the best outcomes are achieved with a whole-food plant-based diet, as well as Mediterranean and vegan diets [111].

Plant fat sources, such as nuts and EVOO, which are rich in unsaturated fats, may improve lipid metabolism and lower TG and low-density lipoprotein (LDL) cholesterol levels. Fiber is also improving cholesterol excretion. In consequence, people who put on plant-based diets have a lower risk of developing hypertension, HF, and other CVD [112]. Cholesterol is found only in animal products, whereas plants contain phytosterols that compete with dietary cholesterol for intestinal absorption, thereby lowering blood cholesterol levels [113]. Moreover, dietary fiber has beneficial effects on gut microbiota, helping to maintain the gut barrier and reducing circulating pro-inflammatory agents [114]. Furthermore, fiber stimulates the secretion of YY peptide and glucagon-like peptide-1 (GLP-1), two hormones that signal satiety and help regulate appetite. Evidence suggests that well-balanced plant-based diets may be beneficial for the therapeutic management and prevention of HFpEF.

### 7.4. Low-Carbohydrate, Ketogenic, and High-Protein Diets

A ketogenic diet is characterized by very low carbohydrate intake (<50 g/day) and a high intake of fats and protein. It may be beneficial for people with T2DM and obesity, but it is not considered optimal for CV and liver health, as demonstrated in animal and human studies. For example, in animal studies, rodents had developed NAFLD and IR while being on a ketogenic diet [115]. The mechanism of this diet is to deplete the body’s glucose reserves, thereby stimulating ketone body production in the liver. Ketone bodies are later used as a backup energy supply for the nervous system [116].

In 2019, a clinical study reported that a two-year ketogenic diet in adults with T2DM improved glycemic control, reduced medication use, lowered body weight and abdominal fat, decreased blood pressure and TG, while elevating liver alanine aminotransferase and HDL and LDL cholesterol [117]. Overall, this diet may benefit patients with diabetes, but caution is advised in those with comorbidities, particularly CVD [117]. The influence of the ketogenic diet on lipid levels depends on its fat composition. In human studies, a ketogenic diet rich in unsaturated fats is associated with a better lipid profile, whereas a high intake of saturated fats is linked with worse blood lipid levels. There is insufficient evidence to determine the effect of the ketogenic diet on blood pressure. Available reports vary and are often contradictory [115].

### 7.5. Sodium Intake, Micronutrients, Vitamins, and the Genome-Based Nutrition

A low-sodium diet is often used in patients with CVD. Hospitalization commonly occurs after a period of high sodium intake [118]. On the other hand, excessively low salt intake can also reduce renal perfusion [100]. The strategy of salt restriction is associated with high readmission rates after cardiac events and even increased mortality, likely because it worsens existing dietary deficits rather than improving overall diet quality [119]. On the contrary, the SODIUM-HF study results did not show a significant difference in hospitalization and mortality rates between groups with reduced dietary sodium intake and those with normal sodium levels [120]. According to Weinberger, only about 25% of normotensive individuals are salt-sensitive, and in the hypertensive population, this percentage ranges from 30% to 50% [121].

Another approach to help those individuals suffering from hypertension is the DASH diet. It combines a low-sodium supply with increased consumption of fiber, protein, and healthy, unsaturated fats. Two clinical studies confirmed the validity of this dietary model: the GOURMET-HF trial [119] and a study by Hummel and colleagues [122].

It has been shown that a high intake of minerals such as potassium, magnesium, and calcium reduces blood pressure in patients with hypertension. Furthermore, high intake of mineral-rich fruits and vegetables has been shown to improve blood pressure and reduce the risk of CHD and stroke [123].

In a 2021 preclinical study, it was demonstrated that NAD^+^ precursors, which are derivatives of vitamin B3, were suggested to improve mitochondrial function and reduce inflammation in HFpEF [124]. In animal and cellular models, all-trans retinoic acid (ATRA) has been suggested to mitigate cardiac remodeling and prevent functional decline in heart failure by counteracting fibrosis, hypertrophy, oxidative stress, and impaired calcium handling [125].

Nowadays, the study of lipid transporter genes requires consideration of differential allele distributions. Hyperglyceridemia and hypercholesterolemia are associated with the ApoE e2 and e4 alleles [126]. Chia, pumpkin seeds, and amaranth are abundant in plant-based MUFA and PUFA and exert anti-inflammatory effects [127].

**Figure 2:**
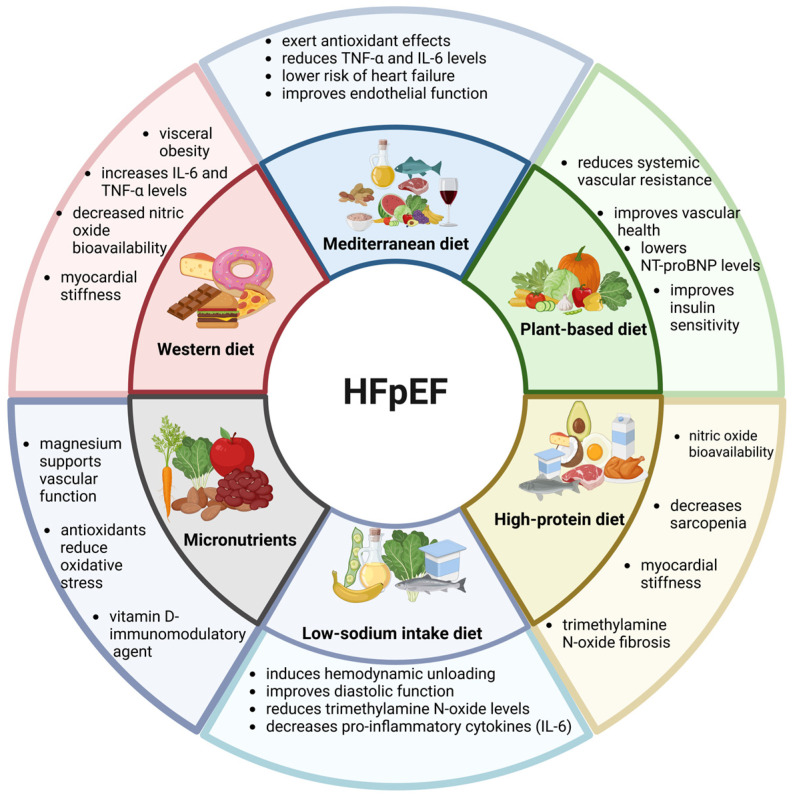
Nutritional Patterns and Their Impact on HFpEF. Created in BioRender. Kalisz, M. (2026) https://BioRender.com/6tpzv4m (accessed on 23 March 2026). Agreement number: HM29IBHE6Y.

In summary, the Western diet promotes metabolic dysfunction, inflammation, and myocardial hypertrophy. In contrast, the Mediterranean and plant-based diets may improve metabolic control, reduce inflammation, and improve myocardial and endothelial function (Figure 2). In contrast, restrictive diets such as ketogenic regimens may benefit glycemic control but require caution in patients with CVD. Supplementing with minerals reduces blood pressure in patients with hypertension. It is important to individualize nutritional strategies in the management of HFpEF.

## 8. Dietary Modulation of Adipose Tissue, Liver, and Immune Function

Overweight and obesity contribute to the development of HFpEF and the progression of the illness [100]. The STEP-HFpEF trial showed that semaglutide improved HF-related symptoms, including physical limitations, exercise function, inflammation, and body weight. It reduced C-reactive protein (CRP) and *N*-terminal pro-B-type natriuretic peptide (NT-proBNP) [128]. Very alarming is the high percentage of patients regaining AT mass after the end of dietary or pharmacological intervention, which leads to the recurrence of the severity of HFpEF. It is even more dangerous, considering that HFpEF patients tend to develop sarcopenia alongside obesity, which further disrupts their metabolism [100].

In patients with obesity phenotype of HFpEF, especially those with excess VAT, sodium retention, and higher aldosterone levels have been observed. Moreover, enhanced leptin receptor signaling may activate the sympathetic nervous system and further mobilize the renin-angiotensin-aldosterone system, thereby additionally upregulating aldosterone secretion. Overactivity of neurohormones leads to increased plasma volume, which in turn causes heart remodeling and fibrosis. All the above mechanisms may promote adipocyte hypertrophy and increase adipogenesis, enhancing the positive feedback loop. The release of leptin and other pro-inflammatory adipokines induces systemic inflammation, thereby promoting fibrosis in the heart and other organs [129].

The role of the gut microbiota should be considered alongside dietary changes. It plays a key role in digestive processes, regulates neurohumoral signaling and gut barrier integrity, and alleviates systemic inflammation. A balanced microbiota prevents diseases, such as T2DM, CVD, obesity, and chronic inflammation [130].

The brain-gut-liver axis communicates via the vagus nerve. It involves the hypothalamus and pituitary gland and plays an important role in the treatment of MetS. Hormones involved in this axis, such as leptin, insulin, GLP-1, ghrelin, and cholecystokinin, regulate glucose homeostasis and hepatic lipid metabolism and reduce appetite. It is important to highlight that insulin is a protein that can partially cross the blood–brain barrier. IR leads to hyperglycemia, which worsens brain health and function, and is involved in glucose neurotoxicity and oxidative stress [130]. Disruption of the gut microbiota results in weakening of the gut barrier, with chemokines and cytokines released to the gut microenvironment that can modulate the function of various immune cells, thereby causing an inflammatory state [130].

The liver is recognized as a center of physiological responses within the gut-brain-liver axis [131]. The liver receives gut-derived signals via the portal veins. An imbalance in the microflora increases gut permeability, allowing toxins to enter the circulation and eading to gastrointestinal conditions such as bloating, constipation, or diarrhea, which further exacerbates systemic inflammation [132].

The Western diet promotes the development of Metabolic-Associated Fatty Liver Disease (MAFLD), with or without fibrosis [133]. In MAFLD, excessive TG accumulation in hepatocytes contributes to hepatic steatosis, chronic inflammation, hepatocyte degeneration, necrosis, and fibrosis [133]. Moreover, the Western diet may disrupt the BAs profile, thereby increasing ROS and further exacerbating inflammation.

## 9. Clinical Implications and Future Therapeutic Strategies

Newer antidiabetic therapies, particularly SGLT2 inhibitors and GLP-1 receptor agonists, have been shown to reduce EAT volume and inflammation while improving CV outcomes, including HFpEF. On the other hand, therapies such as sulfonylureas, thiazolidinediones, and insulin have been associated with increased adipogenesis, EAT accumulation, and a higher risk of HF. SGLT2 inhibitors reduce the risk of HF hospitalization and death, potentially through modulation of EAT, reduction in myocardial inflammation and fibrosis, and improvement of microvascular and hemodynamic function [134,135]. Reduction in EAT accumulation is likely related to decreased overall adiposity and may be partially mediated by the glucose-lowering effects of SGLT2 inhibitors and GLP-1 receptor agonists. A growing body of evidence suggests that the CV effect of GLP-1 and SGLT2 inhibitors may differ [40]. Unlike GLP-1 receptor agonists, which generally show a neutral effect on HF outcomes, SGLT2 inhibitors have consistently been associated with a reduced risk of HF hospitalization.

A growing body of evidence highlights the impact of personalized nutrition in the treatment of HFpEF phenotypes, emphasizing the combination of dietary interventions with standard pharmacotherapy to improve management. As shown in Table 1, each HFpEF phenotype—from cardiometabolic/obesity T2DM to atrial fibrillation, hypertension, ischemia (Coronary Artery Disease (CAD) with Coronary Microvascular Dysfunction (CMD)), chronotropic incompetence, right ventricular/pulmonary and valvular hypertension—has distinct pathophysiological mechanisms and clinical features. Personalized nutritional strategies, such as diets rich in unsaturated fats (MUFA/PUFA), a Mediterranean diet enriched with omega-3 fatty acids, the DASH diet, and optimization of protein/micronutrient intake, can complement pharmacological therapies [128,136,137]. It is hypothesized that this approach may improve the potential of future therapeutic strategies to enhance functional capacity, mitigate the adverse effects of cardiac remodeling, and improve quality of life in patients with HFpEF.

Anti-inflammatory approaches, including inhibition of pro-inflammatory cytokines (e.g., IL-1 and IL-6 pathways), colchicine therapy, statins, and modulation of oxidative stress and inflammasome activation, may also represent a promising strategy for reducing EAT-associated CV risk [138]. In addition, weight-loss interventions, including caloric restriction, exercise programs, or bariatric procedures, have been associated with increased EAT volume and inflammatory activity, leading to improved metabolic and cardiovascular outcomes. It seems that selective reduction in VAT and EAT may be more important than weight loss.

Collectively, these findings suggest that integrating personalized nutrition and exercise with pharmacological therapies, including SGLT2 inhibitors, GLP-1 receptor agonists, and anti-inflammatory agents, may offer synergistic benefits by reducing obesity, EAT, inflammation, and insulin resistance, thereby potentially improving CV outcomes.

## 10. Potential Biomarkers Linking Diet and Organ Crosstalk

HFpEF is often described as a systemic syndrome whose pathophysiology involves complex organ crosstalk between the heart, vascular system, kidneys, liver, adipose tissue, gut, and immune system [139]. Biomarkers that reflect these interactions may facilitate personalized nutritional approaches and help select appropriate therapies for specific HFpEF phenotypes. For example, inflammatory biomarkers such as IL-6 and CRP identify patients more likely to benefit from anti-inflammatory dietary patterns, including Mediterranean or plant-based diets [140]. Evidence from weight-loss–exercise interventions in obese HFpEF demonstrates that reductions in CRP correlate with improvements in peak VO_2_ and functional capacity, supporting the use of inflammatory markers to monitor dietary response [46].

Metabolic biomarkers—including leptin, adiponectin, glycated hemoglobin (HbA1c), ketone concentrations, and indices of IR—aid in stratifying obesity HFpEF phenotypes and predicting benefit from hypocaloric diets, low-glycemic dietary patterns, or unsaturated fatty acid–rich interventions [141,142,143]. In the UFA-Preserved2 trial, increases in circulating MUFA/PUFA biomarkers were associated with trends toward improved peak VO_2_, linking dietary lipid composition to peripheral oxygen utilization and microvascular energetics [144].

Endothelial biomarkers can be used to measure vessel interactions and diet-induced repair. The Mediterranean diet improves flow-mediated vasodilation (FMD), increases endothelial progenitor cells (EPCs), and reduces endothelial microparticles (EMPs), oxidative stress, and eat senescence—changes that directly correlate with improved arterial stiffness and hemodynamic in HFpEF with hypertension or atrial fibrillation [145]. Markers of the oxygen pathway (NO metabolites, nitrate/nitrite balance) have been used to evaluate nitrate supplementation; however, recent meta-analyses have showed no improvement in peak VO_2_ despite modest reductions in Systolic blood pressure (SBP), Diastolic blood pressure (DBP), Mean arterial pressure (MAP), and Systemic vascular resistance (SVR) in HFpEF, limiting their utility as predictive biomarkers [146].

Cardiac remodeling biomarkers, particularly NT-proBNP and galectin-3, provide insight into ventricular–vascular coupling and fibrosis [147]. Although NT-proBNP is often suppressed in obesity, omega-3 fatty acid supplementation has been shown to reduce NT-proBNP levels and improve diastolic parameters in HF, suggesting potential relevance for HFpEF [148].

Gut microbiome-derived metabolites, particularly trimethylamine *N*-oxide (TMAO) and short-chain fatty acids (SCFAs), link dietary quality to endothelial dysfunction and fibrosis, representing targets for dietary modulation or pre-/probiotic therapy [149].

The above biomarkers support integrating dietary interventions with pharmacological management to target phenotype-specific mechanisms in HFpEF.

## 11. Future Directions and Research Gaps

Despite progression in the understanding of HFpEF pathophysiology, there is no single diagnostic biomarker to definitively identify this disease. Natriuretic peptides, including NT-proBNP, remain the most widely used biomarkers in clinical practice. Consequently, there is a growing need to identify new biomarkers of HFpEF that better reflect the complex metabolic, inflammatory, and microvascular mechanisms. A recent study suggests the potential role of soluble suppression of tumorigenicity-2, galectin-3, growth differentiation factor-15, and adipokines (adiponectin, leptin) as markers of myocardial remodeling, inflammation, and adipose tissue dysfunction [150]. However, most of these biomarkers require validation in large prospective cohorts.

The use of dietary biomolecules in HFpEF therapy is promising, but several gaps remain. Future research should integrate proteomics, metabolomics, and metagenomics to identify precise “nutritypes” corresponding to HFpEF phenotype across different populations. Nevertheless, important knowledge gaps remain regarding the reproducibility, longitudinal stability, and clinical applicability of these biomarkers.

Recently, attention has also been focused on biomarkers reflecting interactions between the gut microbiome and CV system. The latest research has identified several potential biomarkers, including gut-derived metabolites (TMAO, SCFAs) that have been associated with systemic inflammation, endothelial dysfunction, myocardial fibrosis, and adverse cardiovascular outcomes. Similarly, circulating EPCs may provide insight into vascular repair capacity and microvascular dysfunction, both recognized contributors to HFpEF pathogenesis. It seems that validating these in large-scale and longitudinal dietary intervention trials will be important.

Another important area of investigation concerns the impact of dietary fatty acids (specifically MUFA and PUFA) on mitochondrial function and oxygen utilization in CVD; however, their mechanistic role in HFpEF remains incompletely understood.

Overall, the complex interplay between diet, adipose tissue biology, gut microbiota, systemic inflammation, and organ crosstalk highlights the need for a systems-biology approach to HFpEF. Combining biomarker discovery with phenotype-specific nutritional and pharmacological interventions may facilitate the development of precision medicine strategies to the specific clinical phenotypes of patients with HFpEF. At present, however, biomarkers in routine clinical practice remain limited by methodological heterogeneity, lack of standardized thresholds, and insufficient prospective validation.

## 12. Conclusions

HFpEF is a multifaceted systemic syndrome that necessitates precision nutrition strategies corresponding to specific phenotypes. The complex crosstalk between the heart, adipose tissue, liver, spleen, and the gut microbiome appears to involve multidimensional biomolecules rather than a single indicator. Different categories of biomarkers associated with inflammatory, metabolic, or endothelial disorders can be used to classify HFpEF into specific phenotypes (e.g., obese vs. aging). This will help target specific dietary interventions to the connection of comorbidities. Evidence suggests that biomarkers such as CRP, HbA1c, and circulating FAs profiles may also provide insight into the biological efficacy of nutritional interventions. Ultimately, integrating nutritional science, CV medicine, and research on organ crosstalk will be important for future HFpEF management.

## Figures and Tables

**Table 1:** Key Comorbidities and Risk Factors influencing individual treatment decisions. A scientific statement from the Heart Failure Association, the European Heart Rhythm Association of the European Society of Cardiology, and the European Society of Hypertension [15].

Key Comorbidities and Risk Factors of HFpEF	The Estimated Prevalence of Important Phenotypes in HFpEF Patients	Outcomes
Arterial Hypertension	60–80%	Associated with a higher risk of death
Elderly (>65 years)	60–70%	Higher morbidity burden (women, white)
CAD	40–70%	Severe hemodynamic impairment is associated with a worse prognosis
Female sex	40–50%	More severe symptoms, poorer quality of life, and lower mortality
Chronotropic Incompetence	30–50%	Associated with reduced tolerance to physical exertion
Obesity	30–40%	More severe symptoms, poorer quality of life, and worse prognosis
Iron Deficiency	20–50%	Lower quality of life and worse prognosis
Sleep Apnea	20–50%	The impact on progression and prognosis of HFpEF is not well-defined
T2DM	20–40%	Poorer quality of life and worse prognosis
Chronic Kidney Disease	20–40%	Increased mortality and complication rates
Atrial FMR	20–40%	Excess mortality
Functional Tricuspid Regurgitation	20–40%	Excess mortality
High Heart Rate (>80 bpm)	20–30%	Related to increased CV risk
Pulmonary Hypertension	20–30%	Severe symptoms and excess mortality
Atrial Fibrillation	15–30%	Higher risk of HF hospitalization due to HF
Cachexia	15–20%	Worse prognosis; increased risk of adverse drug reaction
COPD	15–20%	The safe use of long-acting beta-agonists and muscarinic agonists is not well-defined
Ejection fraction (50–55%)	10–20%	Clinical characteristics and therapeutic response resemble those in HFrEF
Ejection fraction >65%	8–10%	Secondary causes of HFpEF should be considered: amyloidosis and HOCM
Arterial Hypotension	5–10%	Limits the initiation of HF therapies

Abbreviations: COPD, chronic obstructive pulmonary disease; CV, cardiovascular; FMR, functional mitral regurgitation; HF, heart failure; HFrEF, heart failure with reduced ejection fraction; HOCM, hypertrophic obstructive cardiomyopathy; T2DM, type 2 diabetes; CAD, coronary artery disease.

## Data Availability

No new data were created or analyzed in this study. Data sharing is not applicable to this article.

## Abbreviations

The following abbreviations are used in this manuscript:

6MWD	6-Minute walk distance
ACEi	Angiotensin-converting enzyme inhibitors
ACS	Acute coronary syndrome
ARB	Angiotensin receptor blockers
AT	Adipose tissue
ATRA	All-trans retinoic acid
BAs	Bile acids
CAD	Coronary artery disease
CI	Chronotropic incompetence
CIRKO	Mouse model of cardiomyocyte deletion of insulin receptors
CHD	Coronary heart disease
CMD	Coronary microvascular dysfunction
COPD	Chronic obstructive pulmonary disease
CRP	C-reactive protein
CV	Cardiovascular
CVD	Cardiovascular disease
CXCR4	C-X-C Chemokine receptor type 4
DASH diet	Dietary approaches to stop hypertension
DBP	Diastolic blood pressure
DM	Diabetes mellitus
EAT	Epicardial adipose tissue
ECM	Extracellular matrix
EMPs	Reduces endothelial microparticles
EPCs	Increases endothelial progenitor cells
ESC	The European society of cardiology
EVOO	Extra-virgin olive oil
FAs	Fatty acids
FBF	Forearm blood flow
FFAs	Free fatty acids
FGF-21	Fibroblast growth factor-21
FMD	Flow-mediated vasodilation
FMR	Functional mitral regurgitation
FTR	Functional tricuspid regurgitation
FXR	Farnesoid X receptor
GLP-1	Glucagon-like peptide-1
HbA1c	Glycated hemoglobin
HCD	Hypocaloric diet
HDL	High-density lipoprotein
HF	Heart failure
HFpEF	Heart failure with preserved ejection fraction
HFrEF	Heart failure with reduced ejection fraction
HOCM	Hypertrophic obstructive cardiomyopathy
HSC	Hematopoietic stem cell
HYVET	Hypertension in the very elderly trial
IL-1β	Interleukin-1β
IL-6	Interleukins-6
IL-10	Interleukin-10
IL-12	Interleukins-12
IL-23	Interleukin-23
IL-33	Interleukin-33
IR	Insulin resistance
JNK	c-Jun *N*-terminal kinases
KCCQ-CSS	Kansas City cardiomyopathy questionnaire—clinical summary score
LDL	Low-density lipoprotein
LV	Left ventricle
LVEF	Left ventricular ejection fraction
MAFLD	Metabolic-associated fatty liver disease
MAP	Mean arterial pressure
MCP-1	Monocyte chemoattractant protein-1
MetS	Metabolic syndrome
MUFA	Monounsaturated fatty acids
MZ	Marginal zone
NAFLD	Non-alcoholic fatty liver disease
NF-κB	Nuclear factor kappa-light-chain-enhancer of activated B cells
NO	Nitric oxide
NPs	Natriuretic peptides
NT-proBNP	*N*-terminal pro-B-type natriuretic peptide
PALS	Periarteriolar lymphoid sheaths
PAT	Pericardial adipose tissue
PDE5-I	Phosphodiesterase-5 Inhibitors
PH	Pulmonary hypertension
PKG	Protein kinase G
PUFA	Polyunsaturated fatty acids
ROS	Reactive oxygen species
RP	Red pulp
QoL	Quality of life
SAT	Subcutaneous adipose tissue
SBP	Systolic blood pressure
SCFAs	Short-chain fatty acids
SGLT2	Sodium-glucose co-transporter 2
STEP-HFpEF	Agonists semaglutide treatment effect in people with obesity and HFpEF
STEP-HFpEF-DM	Semaglutide treatment effect in people with obesity and HFpEF and diabetes mellitus
SVR	Systemic vascular resistance
T2DM	Type 2 diabetes mellitus
TG	Triglycerides
TGR5	Takeda G protein-coupled receptor 5
TNF-α	Tumor necrosis factor-alpha
TMAO	Trimethylamine *N*-oxide
UFA	Unsaturated fatty acids
WP	White pulp
VAT	Visceral adipose tissue
VCAM-1	Vascular cell adhesion molecule-1
